# The Effects of Escalation of Respiratory Support and Prolonged Invasive Ventilation on Outcomes of Cardiac Surgical Patients: A Retrospective Cohort Study

**DOI:** 10.1053/j.jvca.2019.10.052

**Published:** 2020-05

**Authors:** Vasileios Zochios, Joht Singh Chandan, Marcus J. Schultz, Andrew Conway Morris, Ken Kuljit Parhar, Marc Giménez-Milà, Caroline Gerrard, Alain Vuylsteke, Andrew A. Klein

**Affiliations:** ⁎ University Hospitals Birmingham National Health Service Foundation Trust, Department of Anesthesia and Intensive Care Medicine, Queen Elizabeth Hospital Birmingham, Edgbaston, Birmingham, UK; † Birmingham Acute Care Research Group, Institute of Inflammation and Ageing, Centre of Translational Inflammation Research, University of Birmingham, Birmingham, UK; ‡ University Hospitals of Leicester National Health Service Trust, Department of Anesthesia and Intensive Care Medicine, Glenfield Hospital, Leicester, UK; § Institute of Applied Health Research, University of Birmingham, Birmingham, UK; || Academic Medical Centre (AMC), Amsterdam, The Netherlands; ¶ Mahidol Oxford Tropical Medicine Research Unit (MORU), Bangkok, Thailand; ⁎⁎ Division of Anesthesia, Department of Medicine, University of Cambridge, Addenbrooke's Hospital, Cambridge, UK; †† John Farman Intensive Care Unit, Cambridge University Hospitals National Health Service Foundation Trust, Addenbrooke's Hospital, Cambridge, UK; ‡‡ Department of Critical Care Medicine, University of Calgary, Calgary, Alberta, Canada; §§ Department of Anesthesia and Intensive Care Medicine, Royal Papworth Hospital National Health Service Foundation Trust, Cambridge, UK; |||| Department of Anesthesia and Intensive Care Medicine, Hospital Universitari de Bellvitge, Barcelona, Spain

**Keywords:** cardiac surgery, postoperative pulmonary complications, pulmonary morbidity, invasive ventilation

## Abstract

**Objectives:**

The aim of this study was to determine the effects of escalation of respiratory support and prolonged postoperative invasive ventilation on patient-centered outcomes, and identify perioperative factors associated with these 2 respiratory complications.

**Design:**

A retrospective cohort analysis of cardiac surgical patients admitted to the cardiothoracic intensive care unit (ICU) between August 2015 and January 2018. Escalation of respiratory support was defined as “unplanned continuous positive airway pressure,” “non-invasive ventilation,” or “reintubation” after surgery; prolonged invasive ventilation was defined as “invasive ventilation beyond the first 12 hours following surgery.” The primary endpoint was the composite of escalation of respiratory support and prolonged ventilation.

**Setting:**

Tertiary cardiothoracic ICU.

**Participants:**

A total of 2,098 patients were included and analyzed.

**Interventions:**

None.

**Measurements and Main Results:**

The composite of escalation of support or prolonged ventilation occurred in 509 patients (24.3%). Patients who met the composite had higher mortality (2.9% *v* 0.1%; p < 0.001) and longer median [interquartile range] length of ICU (2.1 [1.0-4.9] *v* 0.9 [0.8-1.0] days; p < 0.0001) and hospital (10.6 [8.0-16.0] *v* 7.2 [6.2-10.0] days; p < 0.0001) stay. Hypoxemia and anemia on admission to ICU were the only 2 factors independently associated with the need for escalation of respiratory support or prolonged invasive ventilation.

**Conclusions:**

Escalation of respiratory support or prolonged invasive ventilation is frequently seen in cardiac surgery patients and is highly associated with increased mortality and morbidity. Hypoxemia and anemia on admission to the ICU are potentially modifiable factors associated with escalation of respiratory support or prolonged invasive ventilation.

A NEW consensus definition of “postoperative pulmonary complications” has been recently proposed by the Standardized Endpoints for Perioperative Medicine (StEP) Collaboration.^1^ This consensus definition consists of 4 rather subjective pulmonary outcome measures, namely atelectasis, pneumonia, aspiration, and the acute respiratory distress syndrome.^1^^,^^2^ The StEP Collaboration also introduced a concept of “severity” of pulmonary complications after surgery, which may reduce the subjectivity of the definition.^1^^,^^2^ In their consensus, severity is classified as “severe” when a patient needs escalation of respiratory support, defined as “unplanned continuous positive airway pressure” (CPAP), “unplanned non-invasive ventilation” (NIV), or “reintubation and invasive ventilation.”^1^

As with other major surgeries, cardiac surgery is associated with postoperative pulmonary morbidity associated with adverse clinical outcomes such as increased mortality and prolonged hospital stay, and also increased healthcare utilization costs.^3^^,^^4^ Postoperative pulmonary complications in the context of cardiac surgery have been poorly defined and cardiac surgery-specific factors such as the use of cardiopulmonary bypass and apnea during cardiopulmonary bypass, intraoperative manipulation of the lungs and thoracic cage, and midline sternotomy appear to increase the risk for pulmonary complications after surgery.^5^^,^^6^

The StEP Collaboration approach has not yet been explored in a cardiac surgical population.^3^^,^^4^ The current study aimed to quantify the rate of escalation of respiratory support (as defined by StEP Collaboration for “severe” pulmonary complications) or prolonged postoperative invasive ventilation (not used by the StEP Collaboration, but yet another frequent and unwanted respiratory complication after cardiac surgery), and to determine their relationship with mortality and morbidity. In addition, perioperative factors predictive of escalation of respiratory support or prolonged invasive ventilation were identified. Establishing the severity of respiratory complications after cardiac surgery, and potentially modifiable risk factors associated with their development, will eventually allow development and evaluation of mitigation strategies. The authors’ null hypothesis was therefore that StEP-defined severe postoperative pulmonary complications and prolonged postoperative invasive ventilation are not associated with adverse outcomes of mortality and intensive care unit (ICU) length of stay.

## Methods

The authors retrospectively examined a cohort of adult cardiac surgical patients who underwent elective cardiac surgery with cardiopulmonary bypass (first-time coronary artery bypass grafting, valve surgery or combined coronary artery bypass with valve surgery) and were admitted to Royal Papworth Hospital National Health Service (NHS) Trust cardiothoracic ICU (a leading heart and lung center in Cambridgeshire, UK, and one of the largest specialist cardiothoracic hospitals in Europe) between August 2015 and January 2018. The study period was selected based on the fact that there were no changes to standard patient management procedures during this period, minimizing a potentially significant source of bias. Patients who underwent redo-sternotomy, postcardiotomy cardiac or respiratory extracorporeal membrane oxygenation, or other procedures (“off pump” surgery, aortic root surgery, heart or lung transplantation, septal defect surgery, and vascular reconstruction) were excluded.

The analysis and reporting adhered to the Strengthening the Reporting of Observational Studies in Epidemiology (STROBE) statement.^7^ The project proposal was reviewed and approved by the Royal Papworth Hospital NHS Foundation Trust Research and Development board (S02402, correspondence March 3, 2018), it was deemed to have no material ethical issues and written informed consent was not a requirement. All data were depersonalized and anonymized.

Data was collected via the perioperative surgical and ICU electronic clinical information systems and the local clinical audit and research data system.

Escalation of respiratory support was defined as described by the StEP Collaboration^1^ as follows:

1. Need for unplanned postoperative use of CPAP, or
2. Need for unplanned postoperative NIV, or
3. Need for reintubation and invasive ventilation.

Prolonged invasive ventilation was defined as need for invasive ventilation beyond 12 hours after surgery.^8^, ^9^, ^10^

Intensive care unit and hospital mortality were defined as death during the time they were in the ICU or in the hospital. Length of stay in ICU was defined as time between point of entry to the ICU to discharge back to the cardiac surgery ward, or time of mortality in ICU if this occurred. Hospital length of stay refers to the day of surgery to the last day in hospital alive.

The local intraoperative and postoperative strategies during the study period were not rigid, but comprised strong advice to use tidal volumes of 6 to 8 mL/kg^–1^ predicted body weight; positive end-expiratory pressure (PEEP) level of 5 cmH_2_O without routine use of alveolar recruitment maneuvers; and cessation of mechanical ventilation and zero PEEP during cardiopulmonary bypass.^11^ The intraoperative red cell transfusion threshold was 70 g/L. Postoperative management in the ICU consisted of cardiac monitoring and optimization of hemodynamics. Weaning of ventilatory support, transition from assist ventilation to spontaneous ventilation, and extubation were conducted when patients met appropriate criteria, namely normothermia, absence of bleeding, established regular spontaneous respiratory pattern, hemodynamic stability, and no residual neuromuscular blockade or abnormal neurologic findings.

Decisions to escalate respiratory support or to continue invasive mechanical ventilation were at the discretion of the attending intensivist. High-flow nasal oxygen therapy was used only seldomly at the time of this study.

Baseline patient characteristics including sex, age, weight, height, body mass index, type of cardiac surgery, logistic, and additive European System for Cardiac Operative Risk Evaluation (EuroSCORE)^12^ were extracted from the electronic clinical information system and the local clinical audit and research data system. Perioperative data (cardiopulmonary bypass time, cross-clamp time, and duration of invasive ventilation) were derived from the local clinical audit and research data system. Hemoglobin levels and ratio of arterial partial pressure of oxygen to inspired fraction of oxygen on admission to ICU were extracted from the electronic clinical information system. Escalation of respiratory support in the first 5 postoperative days, survival, and length of stay data were derived from the local electronic clinical information, clinical audit, and research data systems.

The primary endpoint of the study was the composite of “need for escalation of respiratory support” and “prolonged invasive ventilation.” The composite of postoperative escalation of respiratory support, as defined by StEP collaboration and prolonged invasive ventilation, was chosen as the primary endpoint as it integrates both intraoperative (eg, ventilator-induced lung injury, transfusion-associated lung injury, and transfusion-associated circulatory overload) and postoperative complications (eg, atelectasis); the authors’ composite outcome is therefore a “non-mortality” outcome reflecting quality of perioperative care, which makes it more meaningful to patients, healthcare providers, and the public than specific physiological pulmonary outcomes or individual postoperative pulmonary complications.^1^^,^^2^^,^^13^

Secondary outcomes were the risk of mortality and length of stay in the ICU and hospital. Other outcomes were mediating perioperative factors contributing to the primary outcome.

### Statistical Analysis

Where appropriate, continuous data between groups were compared using either the Student's t test (mean comparison) or Wilcoxon rank sum (median comparison), and categorical data were compared using χ^2^ tests. Where dependent variables were continuous, an adjusted generalized linear regression model was used to assess the impact of a unit of change per dependent variable described as a regression coefficient. Alternatively, where dependent variables were binary an adjusted logistic regression was conducted to assess the unit of change as an odds ratio (OR). The composite outcome consisting of patients who required escalation of respiratory support or prolonged invasive ventilation was described using the frequency of patients rather than treated as separate events to prevent multiple counting of the same individual (eg, to prevent individuals who were intubated for over 12 hours and required postextubation CPAP being counted twice).

Time dependent data, such as “time to extubation” and lengths of ICU and hospital stay, were presented using Kaplan–Meier analyses comparing patients who required escalation of respiratory support or prolonged invasive mechanical ventilation against those who did not require these interventions.

Risk factors were identified contributing to the development of either a requirement for escalation of respiratory support or prolonged invasive ventilation. The models identifying risk factors were developed in accordance with transparent reporting of a multivariable prediction model for individual prognosis or diagnosis guidelines.^14^ Potential risk factors based on demographic and physiological data were prespecified based on a review of the literature and data availability. An unadjusted association between potential risk factors and need for escalation of respiratory support or prolonged invasive ventilation was assessed using univariate logistic regression. A liberal p value threshold of <0.15 was set as the cut-off point after univariate regression to select variables for inclusion in the multiparametric model. Statistical significance in the multivariate model was set at a p value <0.05. Where missing data were present in variables of interest, a complete-case analysis was conducted when developing the regression model, as very few cases had any missing data (n = 9).

After development of a regression model, the multivariate model was internally assessed using bootstrap methods. Each model created was validated on 100 replications using the bootstrap method. These results were then visually compared to the main analysis to assess for any differences in performance.

All analyses were performed using Stata version 14.2 software (StataCorp LLC, College Station, TX). A p value <0.05 was considered statistical significance.

## Results

### Study Population

**Fig 1:**
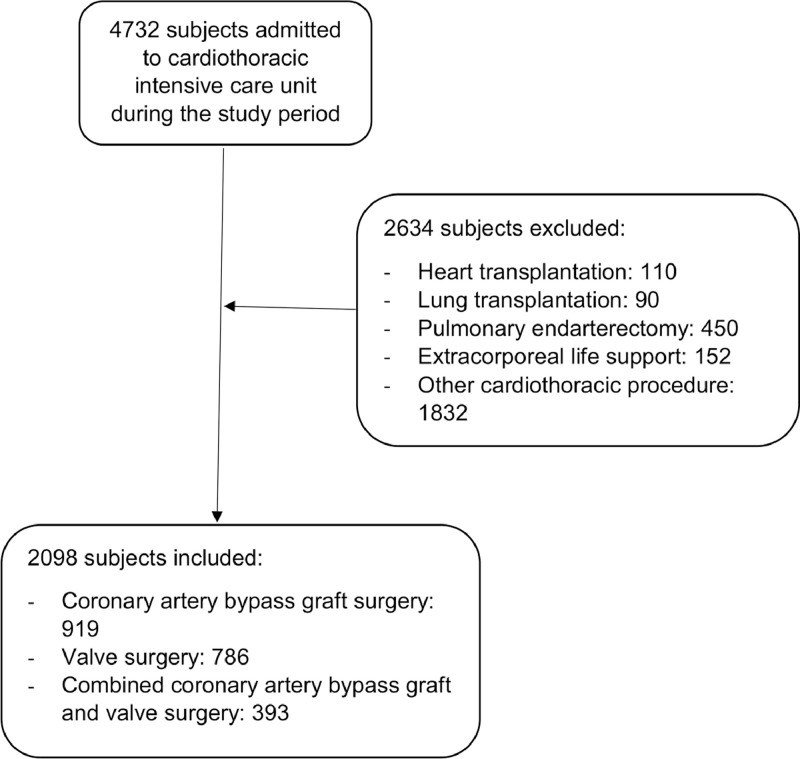
Flow diagram of study population.

Of 4,732 patients admitted, 2,098 patients met the inclusion criteria (Fig 1). Baseline characteristics and outcomes are presented in Tables 1 and 2. The majority of patients was male and underwent coronary artery bypass graft surgery. The median (interquartile range) time to extubation was 6.1 (4.0-11.0) hours.

**Table 1:** Patient Characteristics

Patient Characteristics	Study Cohort (n = 2,098)
Age (y)	69.8 (10.7)
Sex	
Male	1,498 (71.4%)
Height (m)	1.70 (0.10)
Weight (kg)	82 (71-93)
BMI (kg/m^2^)	28.5 (5.2)
Hemoglobin (g/L)	103.4 (15.9)
Logistic EuroSCORE	4.0 (2.1-7.5)
Additive EuroSCORE	5 (3-7)
**Time to extubation** (h)	6.14 (4.04-11.02)
**Surgical characteristics**	
*Type of surgery*	
CABG	919 (43.8%)
Valve surgery	786 (37.5%)
CABG and valve surgery	393 (18.7%)
Cardiopulmonary bypass time (min)	85 (68-105)
Cross-clamp time (min)	57 (44-72)

NOTE. Data are mean (standard deviation), number (%), or median (interquartile range).

Abbreviations: BMI, body mass index; CABG, coronary artery bypass graft; EuroSCORE, European System for Cardiac Operative Risk Evaluation.

**Table 2:** The Occurrence of Escalation of Respiratory Support and Prolonged Invasive Ventilation Within the First 5 Postoperative Days

Outcome	Frequency of Event (n *=* 2,098)	Percentage (%)
CPAP/NIV	126	6.0
Reintubation	40	1.9
Prolonged invasive ventilation (>12 h)	478	23
**Composite outcome groups**		
Escalation of respiratory support (requiring CPAP/NIV or reintubation and invasive ventilation-StEP defined severe pulmonary complications)	154	7.3
Escalation of respiratory support (requiring CPAP/NIV or reintubation and invasive ventilation) and/or prolonged invasive ventilation (>12 h)	510	24.3

NOTE. Data are frequencies of patients experiencing the outcomes and percentages (the composite outcome consisting of patients who required escalation of respiratory support or prolonged invasive ventilation was described using the frequency of patients rather than treated as separate events to prevent multiple counting of the same individual eg, to prevent individuals who were intubated for over 12 hours and required post-extubation CPAP being counted twice).

Abbreviations: CPAP, continuous positive airway pressure; NIV, noninvasive ventilation; StEP, Standardized Endpoints for Perioperative Medicine.

### Escalation of Respiratory Support or Prolonged Invasive Ventilation

Rate of escalation of respiratory support in the first 5 postoperative days was 7.3% and rate of prolonged invasive ventilation was 22.8% (Table 2). The rate of the composite of escalation of respiratory support or prolonged invasive ventilation was 24.3%.

**Fig 2:**
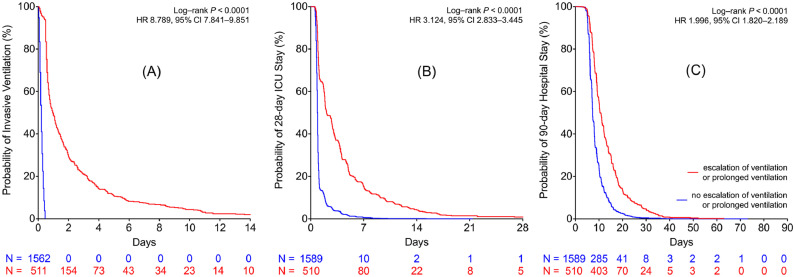
Time to event curves for patients with and without the composite outcome (*Panels A-C*). (A) Time alive whilst receiving invasive mechanical ventilation. (B) Time alive and remaining in intensive care unit. (C) Time alive and remaining in hospital. Escalation of respiratory support (StEP criteria) was defined as unplanned continuous positive airway pressure, noninvasive ventilation or reintubation, and invasive ventilation. Prolonged ventilation was defined as invasive mechanical ventilation for more than 12 hours after exit from operation room. ICU, intensive care unit; StEP, Standardized Endpoints for Perioperative Medicine.

**Fig 3:**
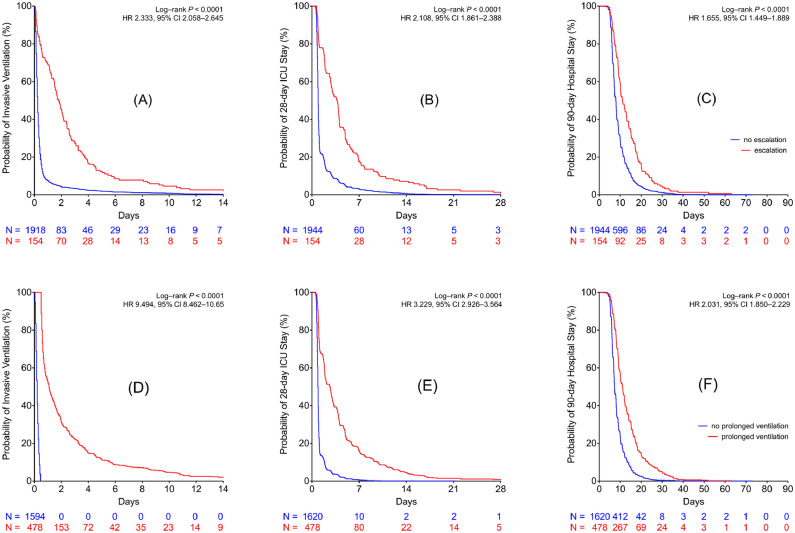
Time to event curves for patients with and without StEP-defined pulmonary complications (*Panels A-C*) and for patients receiving immediate postoperative invasive mechanical ventilation for >12 and <12 hours (*Panels D-F*). (A) and (D) Time alive whilst receiving mechanical ventilation. (B) and (E) Time alive and remaining in intensive care unit. (C) and (F) Time alive and remaining in hospital. Escalation of respiratory support (StEP criteria) was defined as unplanned continuous positive airway pressure, noninvasive ventilation or reintubation, and invasive ventilation. Prolonged ventilation was defined as invasive mechanical ventilation for more than 12 hours after exit from operation room. ICU, intensive care unit; StEP, Standardized Endpoints for Perioperative Medicine.

Patients who met the composite had a longer median time to extubation (23 [14-61] *v* 5 [3-7] hours; p < 0.0001), longer median ICU (2.1 [1.0-4.9] *v* 0.9 [0.8-1.0] days; p < 0.0001) and hospital (10.6 [8.0-16.0] *v* 7.2 [6.2-10.0] days]; p < 0.0001) stay (Table 3, Fig 2). A subgroup analysis is presented in supplementary material section (Fig 3) where the composite group is broken down into patients who required escalation of respiratory support and patients who received prolonged invasive ventilation. “Time to extubation,” “time to discharge from ICU,” and “time to discharge from hospital” was longer in patients with either complication.

**Table 3:** Characteristics of Patients Requiring Escalation of Respiratory Support or Invasive Mechanical Ventilation for More than 12 Hours After Exit From Operation Room Versus the Rest of the Cohort

Variable	Escalation of Respiratory Support_12_ (n *=* 510)	No Escalation of Respiratory Support (n *=* 1,588)	p Value
**Surgical characteristics**			
*Type of surgery*			
CABG	211 (41%)	708 (45%)	
Valve surgery	159 (31%)	627 (40%)	
CABG and valve surgery	140 (28%)	253 (16%)	<0.001
			
Cardiopulmonary bypass time (min)	92.5 (73-120)	84 (66-101.5)	<0.0001
Cross-clamp time (min)	61 (47-82)	56 (44-70)	<0.0001
**Patient characteristics**			
Age (y)	71.5 (10.4)	69.2 (10.8)	<0.0001
Sex			
Male	357 (70%)	1,588 (72%)	
Height (m)	1.69 (0.10)	1.70 (0.10)	0.0989
Weight (kg)	83 (71-97)	81 (71-92)	0.0247
BMI (kg/m^2^)	29.4 (6.0)	28.2 (4.9)	<0.0001
Hemoglobin (g/L) mean (SD)	99.8 (17.2)	104.6 (15.2)	<0.0001
Logistic EuroSCORE	5.1 (2.5-9.2)	3.7 (2.1-6.7)	<0.0001
Additive EuroSCORE	6 (4-8)	5 (3-7)	<0.0001
PaO_2_:F_I_O_2_ ratio	30.5 (22.1-39.0)	35.3 (28.4-42.7)	<0.0001
**Outcomes**			
ICU length of stay (d)	2.1 (1.0-4.9)	0.9 (0.80-1.0)	<0.0001
Hospital length of stay (d)	10.6 (8.0-16.0)	7.2 (6.2-10.0)	<0.0001
Time to extubation (h)	23 (14-61)	5 (3-7)	<0.0001
In-hospital mortality	15 (2.9%)	1 (0.1%)	<0.001

NOTE. Data are mean (SD), number (%), or median (interquartile range). The reported p values were derived from t test (means), Wilcoxon rank sum test (medians), and χ^2^ test (categorical data).

Abbreviations: BMI, body mass index; CABG, coronary artery bypass graft; EuroSCORE, European System for Cardiac Operative Risk Evaluation; ICU, intensive care unit; SD, standard deviation; PaO_2_:F_I_O_2_ ratio, ratio of arterial partial pressure of oxygen to inspired fraction of oxygen.

After adjusting for possible confounding factors, including EuroSCORE, cardiopulmonary bypass time, age, gender, body mass index, cross-clamp time, ICU admission hemoglobin level, admission arterial partial pressure of oxygen to inspired fraction of oxygen ratio, and type of surgery, there was a significant between-group difference in length of ICU stay (regression coefficient 3.0 [95% confidence interval, 1.3-4.8]), hospital length of stay (regression coefficient 10.0 [95% confidence interval, 5.8-14.3]), and in-hospital mortality (2.9% *v* 0.1%; p < 0.001).

### Risk Factors for Escalation of Respiratory Support or Prolonged Invasive Ventilation

The results of the unadjusted univariate logistic regression are summarized in Tables 4 and 5. Additional data on levels of oxygenation and internal validation using bootstrap replication are shown in supplementary material (Appendix A, Supplemental Tables 1-3).^15^ Multivariable adjustment showed that the hemoglobin level (OR 0.98 [0.97-0.99]; p = 0.002) and the arterial partial pressure of oxygen to inspired fraction of oxygen ratio (OR 0.92 [0.90-0.94]; p < 0.001) directly after surgery were significant risk factors for subsequent escalation of respiratory support. These factors remained congruent after bootstrap validation.

**Table 4:** Univariable and Multivariable Regression of Risk Factors for Postoperative Escalation of Respiratory Support (StEP Criteria)

	Univariate Analysis		Included in Multivariate	Multivariate Analysis	
Variable of Interest	Unadjusted odds ratio (95% CI)	p Value	Model (p < 0.15)	Odds ratio (95% CI)	p Value
Age	1.00 (0.99-1.02)	0.669	No	-	-
Sex (male = 1)	1.34 (0.91-1.97)	0.137	Yes	0.98 (0.56-1.70)	0.930
Height	2.52 (0.44-14.42)	0.299	No	-	-
Weight	1.03 (1.02-1.04)	<0.001	Yes	1.01 (0.99-1.03)	0.381
BMI	1.10 (1.07-1.13)	<0.001	Yes	1.03 (0.96-1.11)	0.380
Hemoglobin	0.99 (0.98-1.00)	0.096	Yes	0.98 (0.97-0.99)	0.002
Type of surgery	-	-	Yes	-	-
*CABG*	Ref	Ref	-	Ref	Ref
*Valve surgery*	0.49 (0.33-0.73)	0.001	-	0.67 (0.41-1.09)	0.108
*CABG and valve surgery*	1.03 (0.68-1.55)	0.890	-	0.76 (0.43-1.33)	0.339
Logistic EuroSCORE	1.01 (0.98-1.04)	0.322	No	-	-
Additive EuroSCORE	1.02 (0.96-1.08)	0.625	No	-	-
Cardiopulmonary bypass time	1.01 (1.00-1.01)	<0.001	Yes	1.00 (0.99-1.02)	0.478
Cross-clamp time	1.01 (1.00-1.01)	0.005	Yes	1.00 (0.98-1.02)	0.865
HFNO	0.97 (0.13-7.28)	0.979	No	-	-
PaO_2_:F_I_O_2_ ratio	0.90 (0.89-0.92)	<0.001	Yes	0.91 (0.89-0.93)	<0.001

Abbreviations: BMI, body mass index; CABG, coronary artery bypass graft; CI, confidence interval; EuroSCORE, European System for Cardiac Operative Risk Evaluation; HFNO, high-flow nasal oxygen; PaO_2_:F_I_O_2_ ratio, arterial partial pressure of oxygen to inspired fraction of oxygen ratio; Ref, reference; StEP, Standardized Endpoints for Perioperative Medicine.

**Table 5:** Univariable and Multivariable Regression of Risk Factor for Prolonged Invasive Ventilation

	Univariate Analysis		Included in Multivariate	Multivariate Analysis	
Variable of Interest	Unadjusted Odds Ratio (95% CI)	p Value	Model (p < 0.15)	Odds Ratio (95% CI)	p Value
Age	1.02 (1.01-1.03)	<0.001	Yes	1.00 (0.98-1.01)	0.666
Sex (male = 1)	0.89 (0.71-1.11)	0.284	No	-	-
Height	0.37 (0.13-1.08)	0.069	Yes	0.75 (0.00-303.0)	0.926
Weight	1.01 (1.00-1.01)	0.016	Yes	1.00 (0.94-1.06)	0.967
BMI	1.04 (1.02-1.06)	<0.001	Yes	1.03 (0.87-1.22)	0.726
Hemoglobin	0.98 (0.97-0.99)	<0.001	Yes	0.98 (0.97-0.99)	<0.001
Type of surgery			Yes	-	-
*CABG*	Ref	Ref	-	Ref	Ref
*Valve surgery*	0.88 (0.69-1.11)	0.294	Yes	0.96 (0.68-1.34)	0.796
*CABG and valve surgery*	1.99 (1.53-2.59)	<0.001	Yes	1.10 (0.74-1.65)	0.613
Logistic EuroSCORE	1.06 (1.04-1.08)	<0.001	Yes	1.03 (0.99-1.07)	0.109
Additive EuroSCORE	1.15 (1.10-1.19)	<0.001	Yes	1.08 (0.96-1.21)	0.214
Cardiopulmonary bypass time	1.01 (1.01-1.02)	<0.001	Yes	1.02 (1.01-1.03)	<0.001
Cross-clamp time	1.01 (1.01-1.02)	<0.001	Yes	0.99 (0.98-1.00)	0.032
PaO_2_:F_I_O_2_ ratio	0.96 (0.95-0.97)	<0.001	Yes	0.96 (0.95-0.97)	<0.001

Abbreviations: BMI, body mass index; CABG, coronary artery bypass graft; CI, confidence interval; EuroSCORE, European System for Cardiac Operative Risk Evaluation; PaO_2_:F_I_O_2_ ratio, arterial partial pressure of oxygen to inspired fraction of oxygen ratio; Ref, reference.

## Discussion

Escalation of respiratory support or invasive ventilation beyond 12 hours after cardiac surgery was associated with adverse clinical outcomes of increased mortality and prolonged ICU and hospital length of stay, which are outcomes of interest to patients and relatives, as well as clinicians and healthcare organizations. This was demonstrated in an unselected patient population, which suggests that the StEP collaboration criteria combined with prolonged ventilation are useful for routine surveillance, and may form the metric for quality improvement work in this area. Unsurprisingly, within this cohort, patients undergoing more complex surgery (as defined by longer cardiopulmonary bypass time) and more comorbid patients (higher EuroSCORE) were at higher risk of need for escalation of respiratory support or prolonged invasive ventilation, a finding that adds clinical plausibility to the measure.

It is likely that occurrence of escalation of respiratory support reflects one or more severe postoperative pulmonary complications leading to severe respiratory insufficiency. The StEP Collaboration criteria for severity of respiratory complications after surgery are objective measures that are not susceptible to criteria based on a clinical diagnosis. Indeed, diagnosing pneumonia can be complex, and simple factors such as not using chest radiography routinely or changes in microbiological sampling techniques can alter the reported rates of diagnosis.^16^

One retrospective study of 1,225 cardiac surgical patients found that the rate of unplanned NIV use was 5.1%, which is in line with the authors’ findings (6.0%). However, that study had a smaller sample size and reported reintubation rates in the context of NIV failure only.^17^

The authors demonstrated that hypoxemia and anemia on admission to ICU are associated with escalation of respiratory support or prolonged invasive ventilation. The arterial partial pressure of oxygen to inspired fraction of oxygen ratio has been shown to predict mortality in the cardiac surgical setting.^18^ Its usefulness as a predictor of escalation of respiratory support or prolonged invasive ventilation has not been described before. Perioperative anemia, defined as hemoglobin <100 g/L, leads to a 3-fold increase in risk for postoperative pulmonary complications, independent of type of surgery.^19^^,^^20^ Anemia in the context of cardiac surgery is associated with adverse postoperative outcomes, although in moderate- to high-risk cardiac surgical patients, restrictive transfusion strategies (hemoglobin <75 g/L) are noninferior to liberal transfusion thresholds.^21^, ^22^, ^23^ The causal link between postoperative anemia and respiratory complications after surgery is uncertain. However, one might hypothesize that need for escalation of respiratory support or prolonged invasive ventilation can be explained by higher blood transfusion requirements in anemic patients, potentially resulting in transfusion-related lung injury or circulatory overload.^24^^,^^25^ Of note, it was impossible to include intraoperative ventilatory variables, like PEEP, or other parameters of pulmonary mechanics, such as driving pressure or mechanical power, because these data were not available. Future studies should aim to obtain such data as they may be significant predictors of respiratory complications after surgery, and if so whether they are modifiable.

Several authors have reported outcomes related to prolonged invasive ventilation after cardiac surgery and developed prediction models mainly using 24-, 36-, or 48-hour thresholds for prolonged invasive ventilation.^26^, ^27^, ^28^, ^29^, ^30^, ^31^, ^32^, ^33^ The standard definition of prolonged invasive ventilation according the Society of Thoracic Surgeons is a duration exceeding 24 hours.^13^ It has been shown that “time to extubation” after cardiac surgery longer than 16 hours predicts poor clinical outcomes (morbidity, mortality, and reintubation) and that liberation from the ventilator within the first 9 hours is a predictor of better postoperative outcomes.^34^, ^35^, ^36^ Recent evidence suggests that extubation after 12 hours is associated with poor outcomes and that major morbidity, operative mortality, and prolonged length of stay after cardiac surgery do not significantly increase until “time to extubation” exceeds 12 hours.^8^, ^9^, ^10^ On this basis, here the 12-hour benchmark was incorporated as an indicator of prolonged invasive ventilation into the composite.

In addition to prolonged cardiopulmonary bypass time and aortic cross-clamp time (known risk factors for prolonged invasive mechanical ventilation beyond 24 hours), anemia and hypoxemia on admission to ICU were identified as risk factors for prolonged invasive ventilation.^37^, ^38^, ^39^, ^40^ As an observational study the authors cannot determine the mechanisms that lead to the associations found; however, from the literature the authors can hypothesize that long cardiopulmonary bypass time can lead to pulmonary dysfunction and need for prolonged invasive ventilation through the following mechanisms: systemic inflammatory response and activation of proinflammatory cytokines leading to endothelial damage, increased pulmonary capillary permeability, and extravascular lung water affecting lung compliance and gas exchange.^41^ Similarly, the association between prolonged aortic cross-clamp time and delayed extubation could reflect pulmonary microvascular dysfunction, although the mechanistic link between ischemia-reperfusion and lung injury is not well understood. It is assumed that it is related to an increase in pulmonary vascular resistance and capillary permeability caused by prostaglandins, free radicals, and complement activation.^42^ The association between postoperative anemia and prolonged ventilation may reflect postoperative bleeding and, as a result, maintenance of sedation and invasive ventilation in case reoperation is needed. Hypoxemia may be owing to one or more postoperative pulmonary complications (eg, atelectasis, ventilator-induced lung injury). Hypoxemia would ordinarily delay tracheal extubation until lung tissue is re-recruited and oxygenation is considered adequate.

The strengths of the present study lie in using a large dataset with high level of completeness, the fact that there was no change in practice during the study period, the robust outcome measures with minimal scope for subjectivity, and the excellent follow-up rates. In addition, the authors were able to conduct internal validation of their predictive model using bootstrapping demonstrating internal reliability of their findings.

Certain limitations to this study should be acknowledged. First, its retrospective design renders the study susceptible to selection bias and only routinely recorded data were available, limiting the authors’ ability to analyze factors such as intraoperative ventilation, or report on individual postoperative pulmonary complications (atelectasis, pneumonia, acute respiratory distress syndrome, pulmonary aspiration).^1^^,^^2^ In addition, owing to the observational nature of the study, it was not possible to control for clinical decision-making; however, agreed standards and protocol-driven care minimize variations in individual practice within the institution where this study took place. In the authors’ modeling examining the length of stay, they were unable to account for early mortality in both groups. Although, the number of patients who died during the study period was low (n = 16), this may introduce a possible censoring bias that should be considered in studies. Second, the study was undertaken at a large volume cardiothoracic center, and the risk factor analysis was not externally validated on other data sets; therefore, the authors’ results are not necessarily generalizable nor transportable to other settings or geographic areas. Third, it was not possible to include fluid balance, volumes, and types of transfused blood products, hemodynamic variables, or vasoactive drug data that could potentially be related to risk factors causing planned or unplanned prolonged invasive ventilation (eg, delayed extubation owing to significant hemodynamic instability, hemorrhage, and/or high vasoactive drug requirements or volume overload affecting gas exchange) and escalation of respiratory support (in cases of low cardiac output state and cardiac failure). Finally, other confounding factors such as perioperative respiratory tract infections, heavy smoking history, pre-existing lung disease,^43^ acute onset atrial fibrillation, slow recovery from anesthesia, or acute neurologic deficit potentially could have a hidden effect on the authors’ collapsed composite outcome.

Having validated the StEP criteria for severity of postoperative pulmonary complications in a cardiac surgical population, the authors propose a number of possible uses for this approach. The key question is whether pulmonary complications after surgery are preventable, and if so whether their prevention improves patient-focused outcomes. Potential interventions to test include: early extubation thresholds (eg, 6 or 12 hours), as recent data suggested no detrimental effect of extubation by 6 hours,^8^^,^^10^ perioperative oxygenation targets, effect of perioperative transfusion strategies and intraoperative ventilatory strategies, including PEEP and alveolar recruitment maneuvers. If the authors’ risk adjustment is validated in subsequent studies, it may offer a method for producing risk-adjusted postoperative pulmonary complication rates allowing effective prospective comparison within and between units, facilitating the use of postoperative pulmonary complications rates as a quality measure.

## Conclusion

In a low- to medium-risk patient population undergoing routine cardiac surgery, escalation of respiratory support or prolonged invasive ventilation are associated with adverse outcomes. Hypoxemia and anemia after cardiac surgery are potentially modifiable risk factors for pulmonary complications, which need to be addressed better in future studies.

## Appendix Supplementary materials

Image, application 1
