# Supplementary material for: The Effects of Escalation of Respiratory Support and Prolonged Invasive Ventilation on Outcomes of Cardiac Surgical Patients: A Retrospective Cohort Study
Source: J Cardiothorac Vasc Anesth. 2020 May;34(5):1226–34. doi: 10.1053/j.jvca.2019.10.052 (PMC7144337; doi:10.1053/j.jvca.2019.10.052)
Supplement: Supplementary file 1 [file mmc1.docx]

**Supplemental Table 1**. Initial postoperative arterial partial pressure of oxygen to inspired fraction of oxygen ratios on admission to intensive care unit.

|  | **N patients** | **%** | **Mean P_a_O_2_:F_i_O_2_ ratio (SD)** | **Median P_a_O_2_:F_i_O_2_ ratio (IQR)** |
| --- | --- | --- | --- | --- |
| **Total** | 2098 | 100 | 263.3 (84) | 258 [203.3 to 315] |
| **No/mild hypoxemia (mmHg)^a^** | 1599 | 76.2 | 294.8 (1.7) | 283.5 [242.3 to 335.3] |
| **Moderate/severe hypoxemia (mmHg)^a^** | 490 | 23.4 | 161.3 (25.5) | 165 [143.3 to 183] |
| Missing data | 9 | 0.4 |  |  |

Data are mean (SD), number (%) or median [interquartile range].

Abbreviations: P_a_O_2_:F_i_O_2_ ratio, ratio of arterial partial pressure of oxygen to inspired fraction of oxygen; SD, standard deviation.

^a^ Hypoxemia severity defined according to Berlin criteria for acute respiratory distress syndrome.^15^

**Supplemental Table 2**. Internal validation using bootstrap replication for escalation of respiratory support multivariate model.

| **Variable of interest:**  **Escalation of respiratory support** | **Observed odds ratio (95% CI)** | ***P* value** |
| --- | --- | --- |
| Sex (Male=1) | 0.98 (0.55 to 1.74) | 0.933 |
| Weight | 1.01 (0.99 to 1.03) | 0.350 |
| BMI | 1.03 (0.97 to 1.10) | 0.323 |
| Hemoglobin | 0.981 (0.97 to 0.99) | 0.001 |
| Type of surgery |  |  |
| CABG | Ref | Ref |
| Valve surgery | 0.67 (0.39 to 1.16) | 0.149 |
| CABG and valve surgery | 0.76 (0.46 to 1.27) | 0.297 |
| Cardiopulmonary bypass time | 1.00 (0.99 to 1.02) | 0.613 |
| Cross clamp time | 1.00 (0.98 to 1.03) | 0.901 |
| P_a_O_2_:F_i_O_2_ ratio | 0.91 (0.88 to 0.95) | <0.001 |

Abbreviations: BMI, body mass index; CABG, coronary artery bypass graft; CI, confidence interval; P_a_O_2_:F_i_O_2_ ratio, arterial partial pressure of oxygen to inspired fraction of oxygen ratio; Ref, reference

**Supplemental Table 3**. Internal validation using bootstrap replication for the prolonged invasive ventilation multivariate model.

| **Variable of Interest**  **Invasive mechanical ventilation for > 12 hours** | **Observed odds ratio (95% CI)** | ***P* value** |
| --- | --- | --- |
| Age | 1.00 (0.98 to 1.01) | 0.662 |
| Sex (Male=1) |  |  |
| Height | 0.75 (0.00 to 395.5) | 0.929 |
| Weight | 1.00 (0.94 to 1.06) | 0.968 |
| BMI | 1.03 (0.87 to 1.22) | 0.730 |
| Hemoglobin | 0.98 (0.97 to 0.99) | <0.001 |
| Type of surgery |  |  |
| *CABG* | Ref | Ref |
| *Valve surgery* | 0.96 (0.68 to 1.34) | 0.796 |
| *CABG and valve surgery* | 1.10 (0.74 to 1.66) | 0.621 |
| Logistic EuroSCORE | 1.03 (0.99 to 1.07) | 0.115 |
| Additive EuroSCORE | 1.08 (0.96 to 1.21) | 0.226 |
| Cardiopulmonary bypass time | 1.02 (1.01 to 1.03) | <0.001 |
| Cross clamp time | 0.99 (0.98 to 1.00) | 0.020 |
| P_a_O_2_:F_i_O_2_ ratio | 0.96 (0.95 to 0.98) | <0.001 |

Abbreviations: BMI, body mass index; CABG, coronary artery bypass graft; CI, confidence interval; EuroSCORE, European System for Cardiac Operative Risk Evaluation; P_a_O_2_:F_i_O_2_ ratio, arterial partial pressure of oxygen to inspired fraction of oxygen ratio; Ref, reference
